# Breaking silence, igniting action: Noma's wail for global health recourse and intervention

**DOI:** 10.1002/hsr2.2083

**Published:** 2024-05-27

**Authors:** Patrick Ashinze, Mafua Nelson, Aremu Sikiru Ademola, Eniola Akande, Frederick Oyekunle Moody, Bethrand Ozioma Chukwu, Olajuwon Tolulope Joseph, Andrew Awuah Wireko, Toufik Abdul‐Rahman

**Affiliations:** ^1^ 82 Division Medical Service Hospital Enugu Nigeria; ^2^ Faculty of Clinical Sciences, University of Ilorin Ilorin Nigeria; ^3^ Division of Research Toufik's World Medical Association Sumy Ukraine; ^4^ Faculty of Clinical Sciences, Madonna University Ogene Anambra Nigeria; ^5^ RMO International Worcestershire UK; ^6^ Medical Institute, Sumy State University Sumy Ukraine

**Keywords:** Africa, global health, neglected tropical diseases, noma

Dear Editor,

This correspondence is written to draw your attention to the recent announcement by the World Health Organization (WHO) designating noma (cancrum oris) as a Neglected Tropical Disease (NTD).^1^ This significant development merits careful consideration due to its potential implications for public health, particularly in Africa and beyond.

The WHO's decision to classify noma as an NTD underscores the pressing need for increased research, funding, and intervention strategies to address this neglected health issue. In Africa, where noma primarily affects children in impoverished communities, the recognition of noma as a neglected disease brings attention to the broader challenges faced by the continent in combating neglected diseases. It highlights the critical importance of strengthening healthcare systems, enhancing disease surveillance, and fostering international collaboration to ensure a comprehensive response to NTDs.

Noma, also called oro‐facial gangrene or cancrum oris, is a severely debilitating disease that predominantly affects malnourished children between the ages of 2 and 7.^2^ It is characterized by rapid progression from intraoral inflammation to grotesque cutaneous necrosis and gangrene. The key risk factors for noma include multidimensional poverty(principal risk factor), malnutrition, poor oral hygiene, and chronic infections.^1^, ^2^

Based on existing studies, the global incidence and prevalence figures of noma are outdated, are at best educated guesses, and are not based on broad epidemiological studies.^3^

Patients with noma have been diagnosed in at least 23 countries in the past decade, with most cases reported from a few countries.^2^, ^3^

Commonly associated conditions with noma include malnutrition, gastroenteritis, measles, malaria, and anemia. The clinical sequelae of no ma often requires complex surgical reconstruction to relieve ankylosis and to replace lost tissue.^2^ The occurrence of fresh noma is closely linked to linear growth retardation seen in deprived children aged 3–30 months.^3^

Fresh noma is likely programmed in early life due to malnutrition and chronic infections like HIV.^4^

The management of acute noma requires antibiotics and nutritional support to save the child's life. Multidisciplinary teams may not be available to families living in poverty in remote areas. When the child is stable and if transfer to a tertiary center is possible, referral is recommended.

Wound care entails topical irrigation with hydrogen peroxide, saline, and 0.2% chlorhexidine to deslough the necrotic tissue and toothbrushing to remove dento‐gingival bacterial plaque.^5^ Once the acute phase of the necrotizing disease has been controlled, debridement of roots, necrotic soft tissue, and necrotic bone is carried out.^5^, ^6^ Judicious use of analgesia, adequate hydration, correction of electrolytes, and vitamin deficiencies with nutritional support is essential. Antibiotics (metronidazole, amoxicillin) will arrest or retard the progression into septicemia.^6^

Late‐stage presentation may require extensive plastic/reconstructive surgery.^4^, ^6^

The economic and social effects of NTDs especially noma in Sub‐Saharan Africa are an increase in vulnerable age group mortality as it affects the extremely poor who cannot afford the high cost of care thereby aggravating their fates with a profoundly high mortality rate in untreated patients.^7^ It causes an increase in productive labor loss due to functional impairments in productive age groups, consequently causing a negative psychological affectation likewise.^6^, ^7^ Noma also causes unsightly facial disfigurements, leading to social alienation and worsening mental health. Its significantly expensive rehabilitative care also increases pressure on public and private donor funding.^7^

As researchers and public health advocates, it is imperative that we delve into the potential socio‐economic consequences of noma and the broader implications of neglecting diseases that disproportionately impact vulnerable populations. Addressing noma requires not only medical interventions but also a holistic approach that considers social determinants, cultural factors, and systemic inequalities.

Furthermore, this designation prompts us to reflect on the role of global health initiatives and their responsiveness to emerging challenges. How can the international community mobilize resources effectively to tackle noma and other neglected diseases like trachoma, leprosy, and schistosomiasis? What lessons can we learn from successful interventions against the other NTDs?

In conclusion, the recognition of noma as a NTD by the WHO is a call to action for the scientific community, policymakers, government leaders, and healthcare practitioners. Being a disease characterized by rapid progression and disfigurement, it would require comprehensive primary healthcare, including prevention through improved nutrition and hygiene. Also, a comprehensive study of the global prevalence should be carried out for epidemiological data collation, analysis, and storage. This therefore gives an opportunity to unite efforts, share knowledge, and implement evidence‐based strategies to alleviate the suffering caused by noma and prevent its further spread. We encourage the research community to contribute actively to this dialogue, fostering a collaborative and multidisciplinary approach to address the challenges posed by noma on both local, regional, and global scales.

## AUTHOR CONTRIBUTIONS

**Patrick Ashinze**: Conceptualization; validation; writing—original draft. **Mafua Nelson**: Writing—original draft. **Aremu Sikiru Ademola**: Writing—original draft. **Eniola Akande**: Writing—original draft. **Frederick Oyekunle Moody**: Writing—original draft. **Bethrand Ozioma Chukwu**: Writing—original draft. **Olajuwon Tolulope Joseph**: Writing—original draft. **Andrew Awuah Wireko**: Writing—review & editing. **Toufik Abdul‐Rahman**: Writing—review & editing; supervision.

## CONFLICT OF INTEREST STATEMENT

The authors declare no conflicts of interest.

## TRANSPARENCY STATEMENT

The lead author Toufik Abdul‐Rahman affirms that this manuscript is an honest, accurate, and transparent account of the study being reported; that no important aspects of the study have been omitted; and that any discrepancies from the study as planned (and, if relevant, registered) have been explained.

## Data Availability

Data sharing is not applicable to this article as no new data were created or analyzed in this study. No new data was generated.
